# Supplementary material for: Structure of phospholipase Cε reveals an integrated RA1 domain and previously unidentified regulatory elements
Source: Commun Biol. 2020 Aug 14;3:445. doi: 10.1038/s42003-020-01178-8 (PMC7427993; doi:10.1038/s42003-020-01178-8)
Supplement: Supplementary file 1 — Supplementary Information [file 42003_2020_1178_MOESM1_ESM.pdf]

SUPPORTING INFORMATION FOR

Structure of phospholipase Cε reveals an integrated RA1 domain and novel regulatory elements

Ngango Y. Rugema[1†], Elisabeth E. Garland-Kuntz[1†], Monita Sieng[1], Kaushik Muralidharan[2], Michelle Van Camp[1], Hannah O'Neill[1], William Mbongo[2], Arielle F. Selvia[1], Andrea T. Marti[2], Emmanda McKenzie[2], Amanda Everly[1], and Angeline M. Lyon[1,2*]

From the [1]Department of Chemistry and the [2]Department of Biological Sciences, Purdue University, West Lafayette, IN 47907

*To whom correspondence should be addressed: Angeline M. Lyon, Departments of Chemistry and Biological Sciences, Purdue University, 560 Oval Drive, West Lafayette, Indiana 47907, Telephone: (765)-494-5291; email: lyonam@purdue.edu

[†]Authors contributed equally to the work.

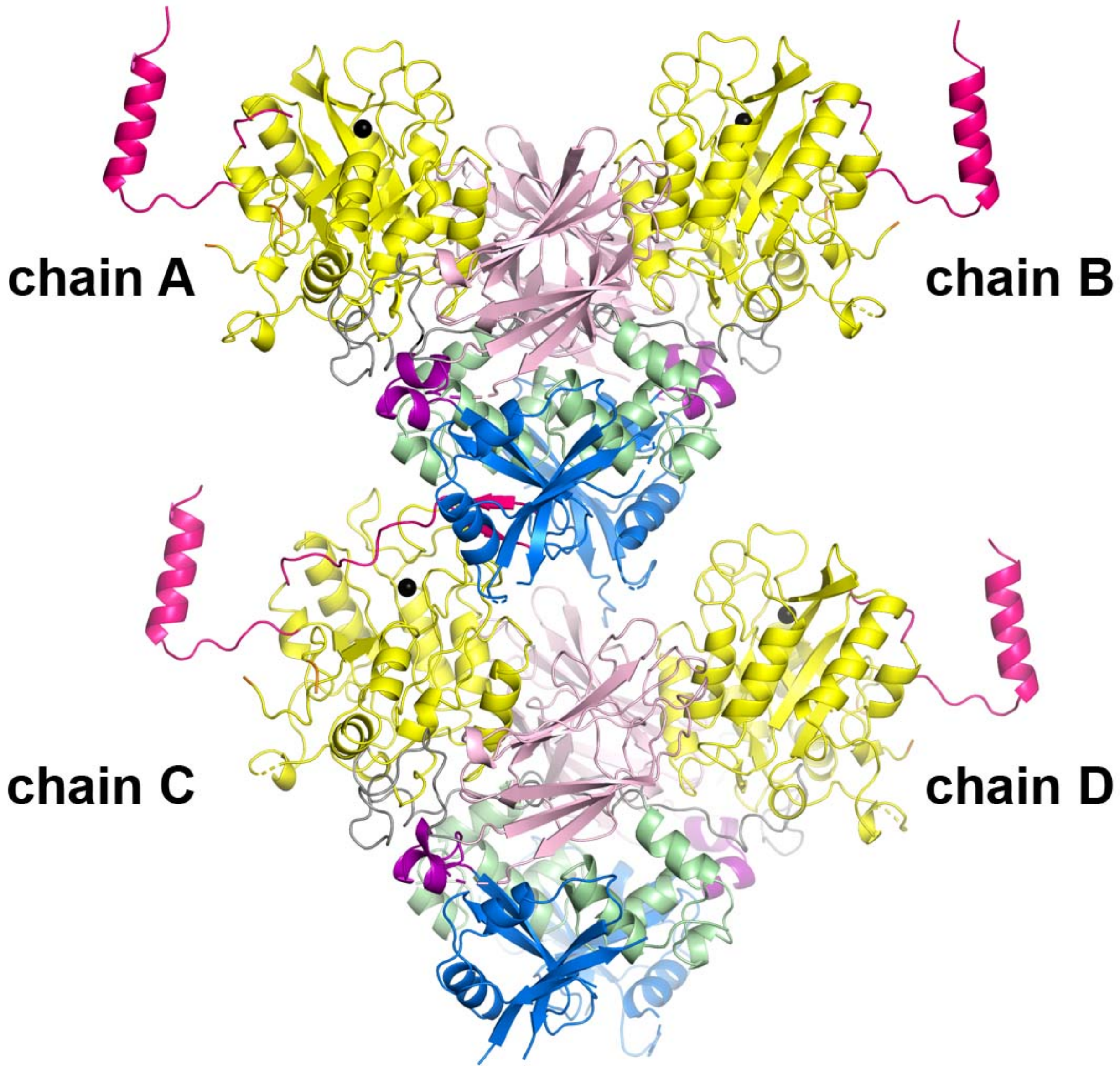

Supplementary Figure 1. The asymmetric unit contains four copies of PLCε EF3-RA1. Within the asymmetric unit, chains A and B (top) and chains C and D (bottom) form dimers through their TIM barrel–EF3/4 interfaces. Domains are colored as in Figure 1A. F_o-F_c difference Fourier maps generated peaks consistent with Ca^{2+} (black sphere) in each of the four chains, All four chains were similar, with an r.m.s.d. of 0.206 – 0.223 Å.

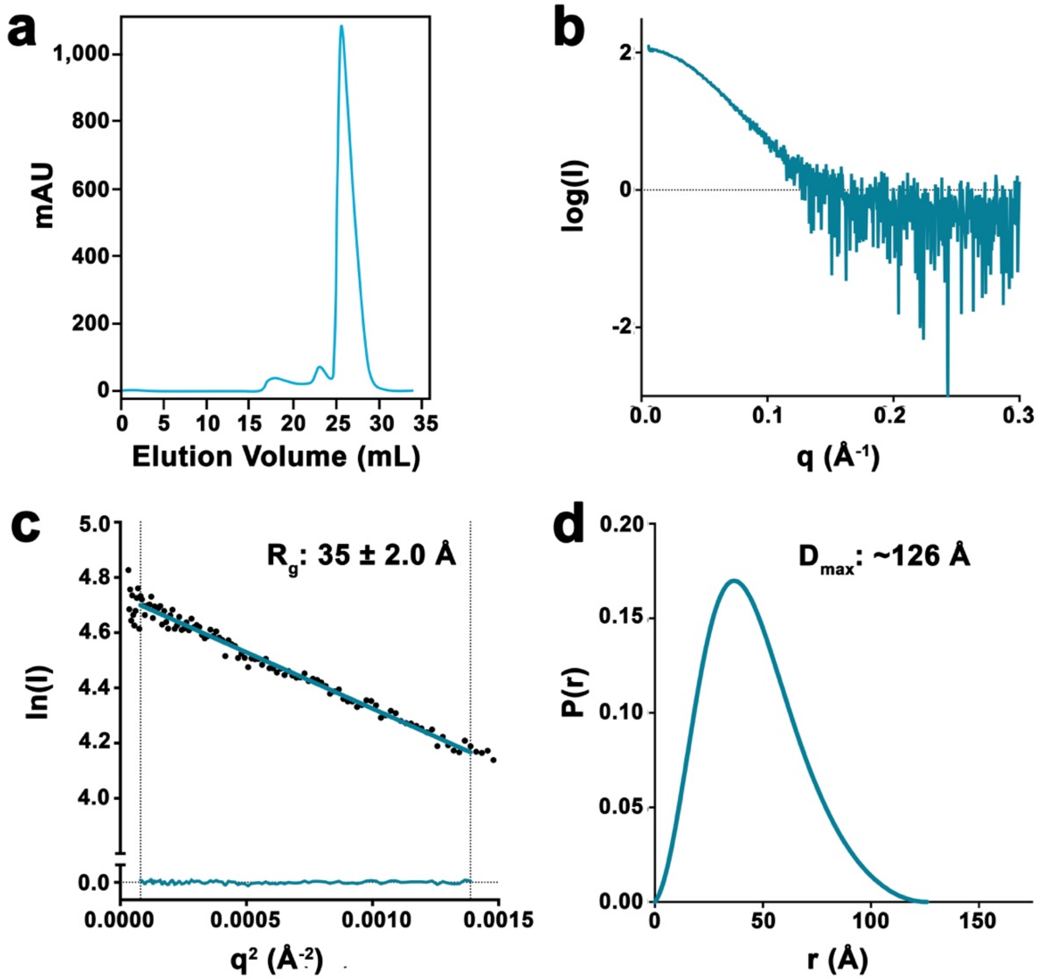

Supplementary Figure 2. PLCε EF3-RA1 is primarily monomeric and monodisperse in solution. (A) Size exclusion chromatogram showing the elution profile of PLCε EF3-RA1. The (B) raw scattering curve, (C) Guinier plot, and (D) pair-distance distribution function are consistent with a largely globular protein that is monomeric in solution[22].

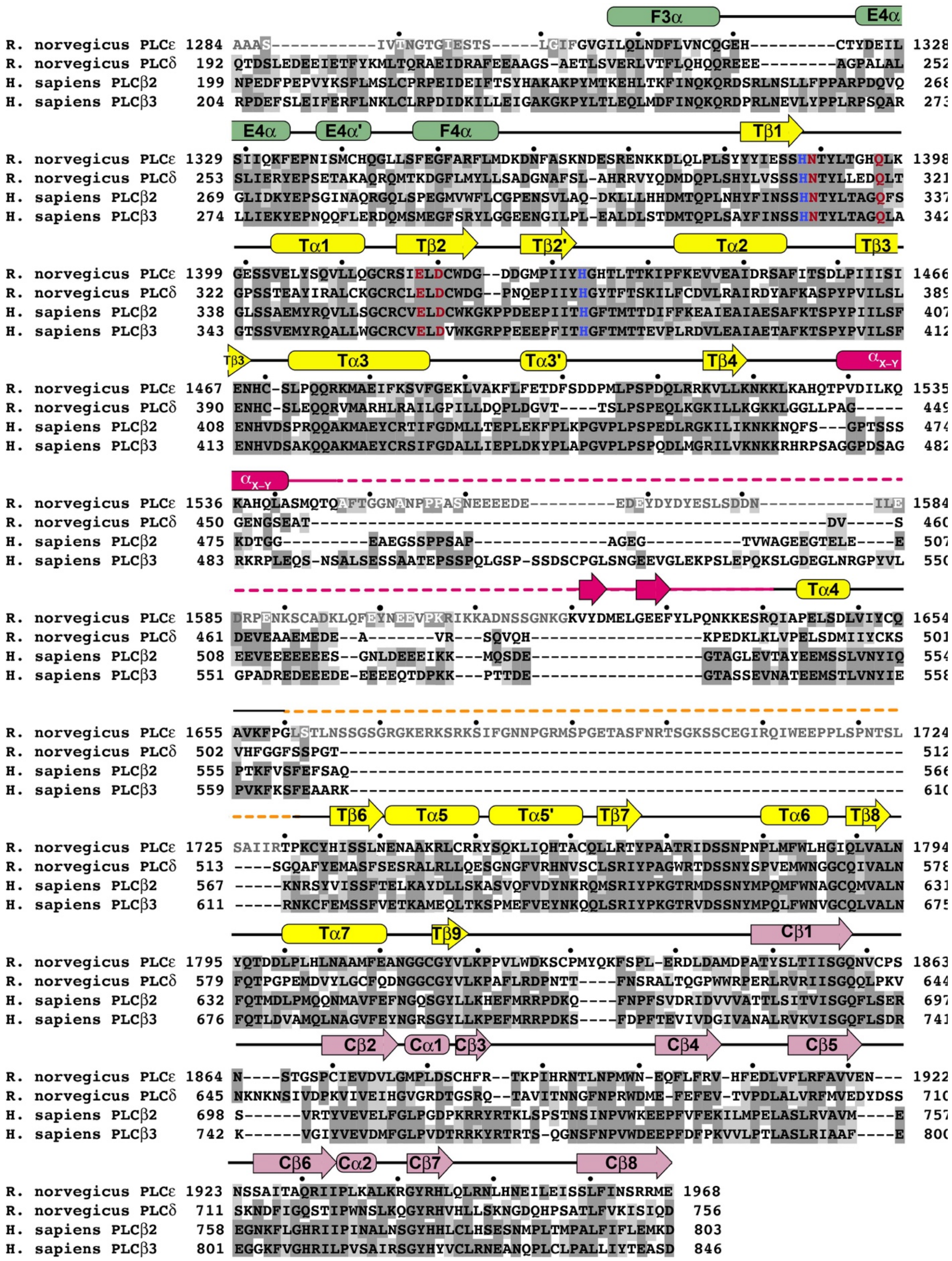

Supplementary Figure 3. Sequence and secondary structure alignment of phospholipase C enzymes. The amino acid sequences of *R. norvegicus* PLCε (UNIPROT Q99P84), *R. norvegicus*

PLCδ (UNIPROT P10688), *H. sapiens* PLCβ2 (UNIPROT Q00722), and *H. sapiens* PLCβ3 (UNIPROT Q01970) spanning the EF3-C2 domains are shown. Sequence identity is highlighted in dark gray, and sequence similarity is highlighted in light gray. The observed secondary structure is shown above the alignment, with β strands shown as arrows, α helices as rounded rectangles, and disordered regions as dashed lines. The secondary structure elements are referred to as described in the structure of *R. norvegicus* PLCδ[23], with the exception of the α_{X-Y} helix. PLCε residues shown in gray or white are disordered in the crystal structure. Residues in red are involved in coordinating the catalytic Ca^{2+} ion, and residues in blue correspond to catalytic histidines[2]. The black circles above the alignment are spaced every ten amino acids for reference.

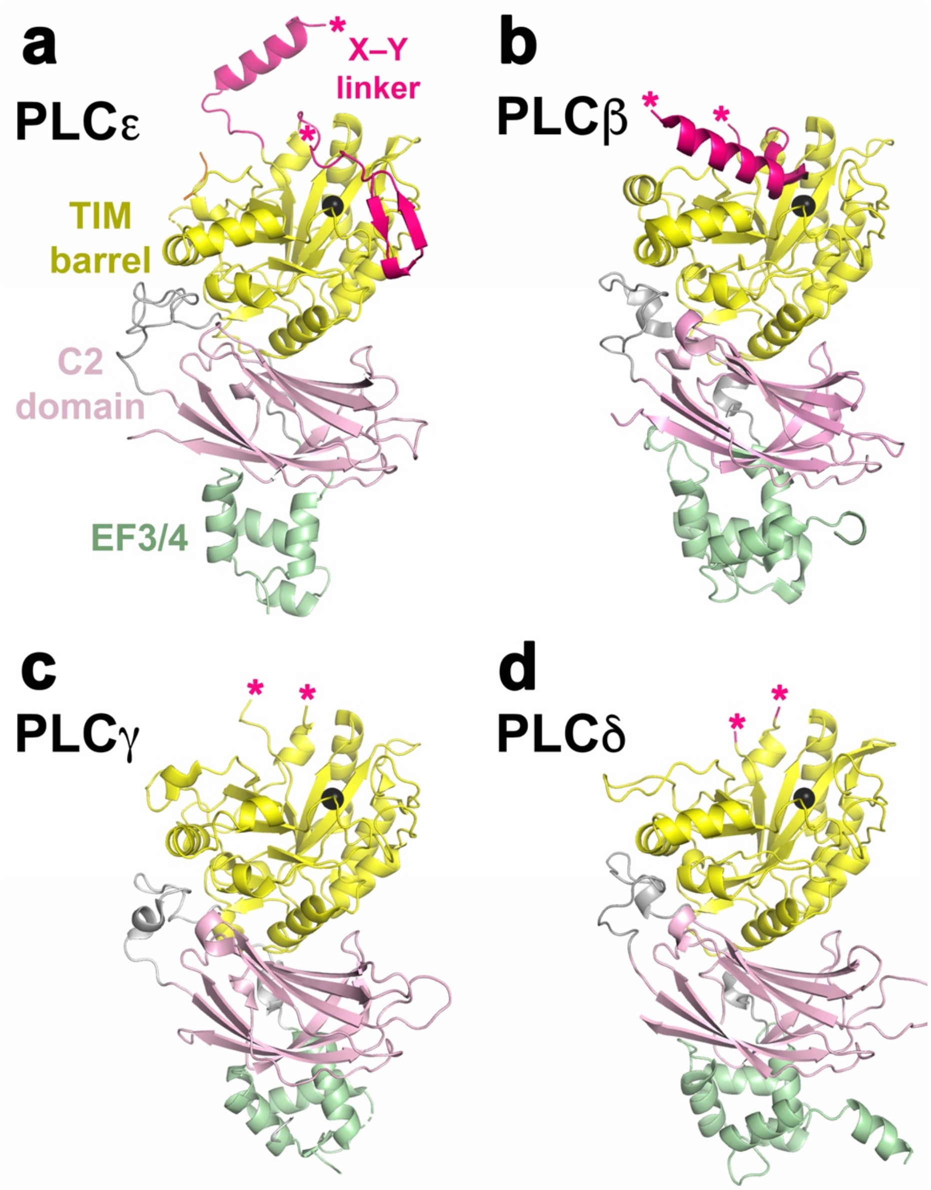

Supplementary Figure 4. **Comparison of the PLCε EF3-C2 core domains across PLC subfamilies.** The crystal structures of the EF3-C2 domains from (A) *R. norvegicus* PLCε (PDB ID 6PMP, this work), (B) *H. sapiens* PLCβ3 (PDB ID 3OHM[39]), (C) *R. norvegicus* PLCγ1 (PDB ID 6PBC[26]), and (D) *R. norvegicus* PLCδ1 (PDB ID 2ISD[23]). Domains are colored as in (A), and the catalytic Ca^{2+} is shown as a black sphere. Asterisks indicate the first and last ordered residues within the X–Y linker (hot pink) in PLCε, PLCβ, and PLCδ, or the ends of the regulatory domains in PLCγ.

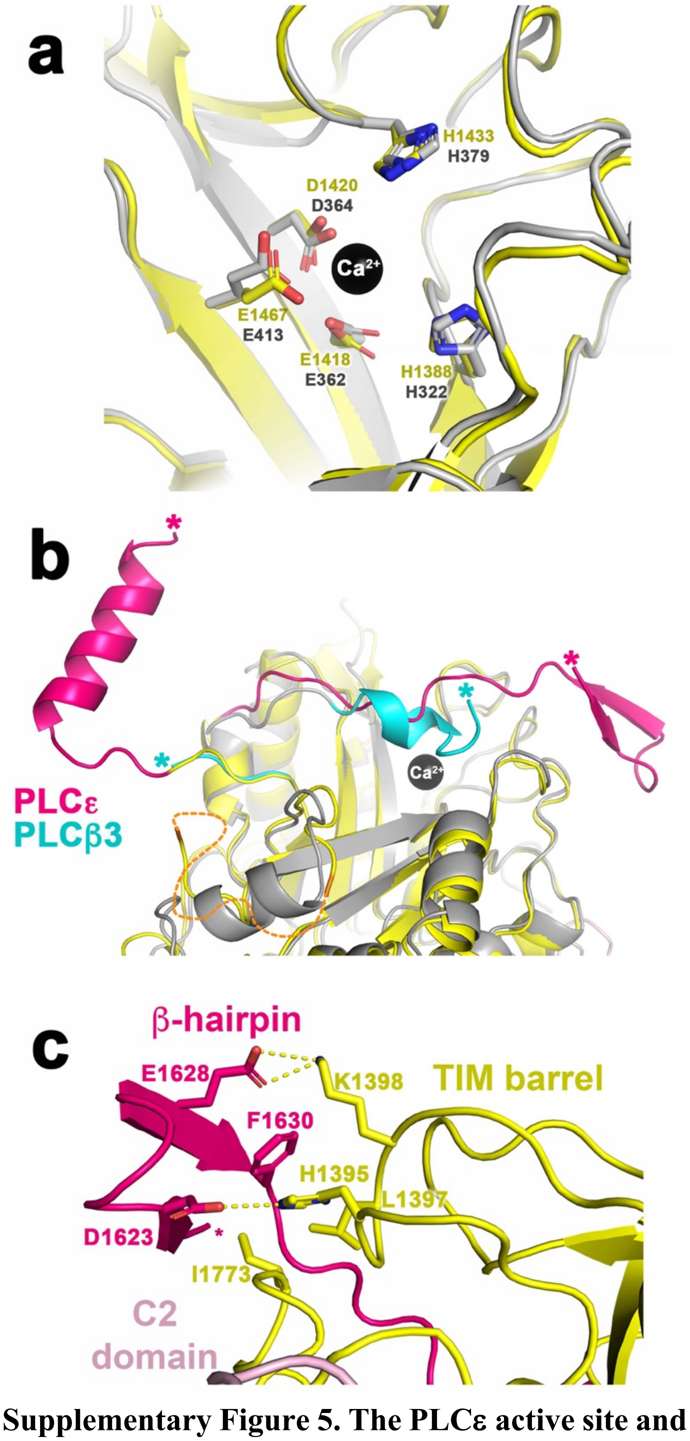

Supplementary Figure 5. The PLCε active site and X–Y linker share similarities with PLCβ. (A) The architecture of the PLCε EF3-RA1 active site (yellow) is conserved with the PLCβ3 active site (gray, PDB ID 3OHM[39]). The acidic residues shown help coordinate the active site Ca^{2+} (black sphere), along with catalytic histidines. (B) The PLCε EF3-RA1 linker was observed to form the $\alpha_{X\text{–}Y}$ helix and a β-hairpin. This shares some similarity to the "lid helix" in PLCβ3 (in blue), which must be displaced prior to substrate binding. (C) Interactions of the X–Y linker β hairpin with residues on the surface of the TIM barrel. Dashed yellow lines indicate hydrogen bonds or salt bridges ≤ 3.5 Å.

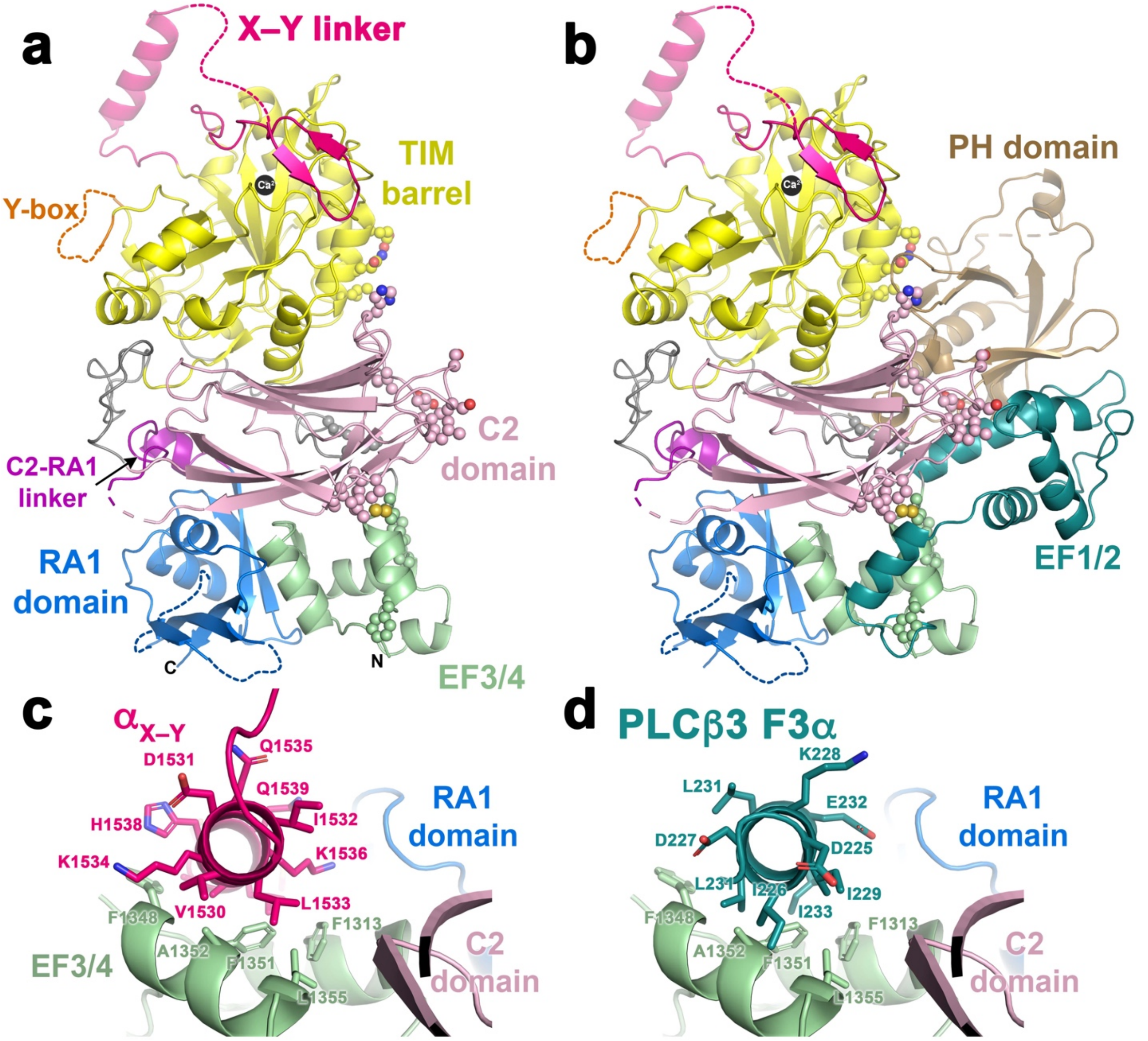

Supplementary Figure 6. The structure of PLCε EF3-RA1 is compatible with the predicted location of the PH and EF1/2 domains. (A) Conserved residues on the surface of EF3/4 and the TIM barrel and C2 domains that could form canonical interactions with the PH domain and EF1/2 domains in the context of the complete PLCε core structure instead mediate crystal contacts. The side chains of these residues are shown as balls and sticks, and domains colored as in Figure 1A. (B) Superimposing the PLCβ3 EF3-C2 domains (PDB ID 3OHM[39]) with those of PLCε shows the structure is compatible with the canonical positions of the PH domain, EF1/2, and the F3α helix of EF3, as seen in other PLC structures. The r.m.s.d. for the PLCβ3 and PLCε EF3-C2 domains is 1.079 Å for 355 Cα atoms. (C) In the PLCε EF3-RA1 structure, the hydrophobic face of the α_{X-Y} helix interacts with a hydrophobic surface on EF3/4 through an *in trans* crystal contact. In the presence of the PH domain, EF1/2, and the F3α helix, (shown in B), the EF3/4 surface interacts with the F3α helix, which is disordered in the EF3-RA1 structure.

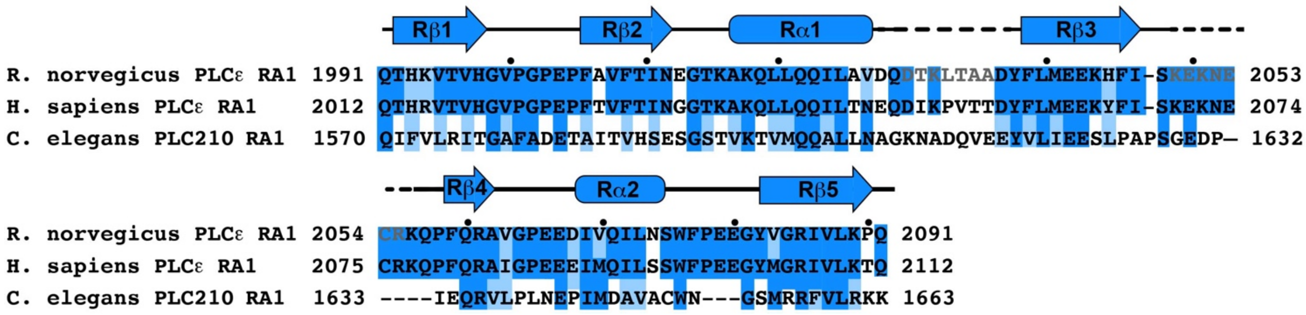

Supplementary Figure 7. Sequence and secondary structure alignment of the PLCε RA1 domain. The amino acid sequences of the RA1 domain from *R. norvegicus* PLCε (UNIPROT Q99P84), *H. sapiens* PLCε (UNIPROT Q9P212), and *C. elegans* PLCε (UNIPROT G5EFI8) are shown. Sequence identity is highlighted in royal blue, and sequence similarity is highlighted in light blue. The secondary structure of the domain, based on the PLCε EF3-RA1 structure and the solution structure of the RA1 domain (PDB ID 2BYE[18]), is shown above the sequence. β strands shown as arrows, α helices are shown as rounded rectangles, and disordered regions are shown as a dashed line. Residues in gray are disordered in the crystal structure. The black circles above the alignment are spaced every ten amino acids for reference.

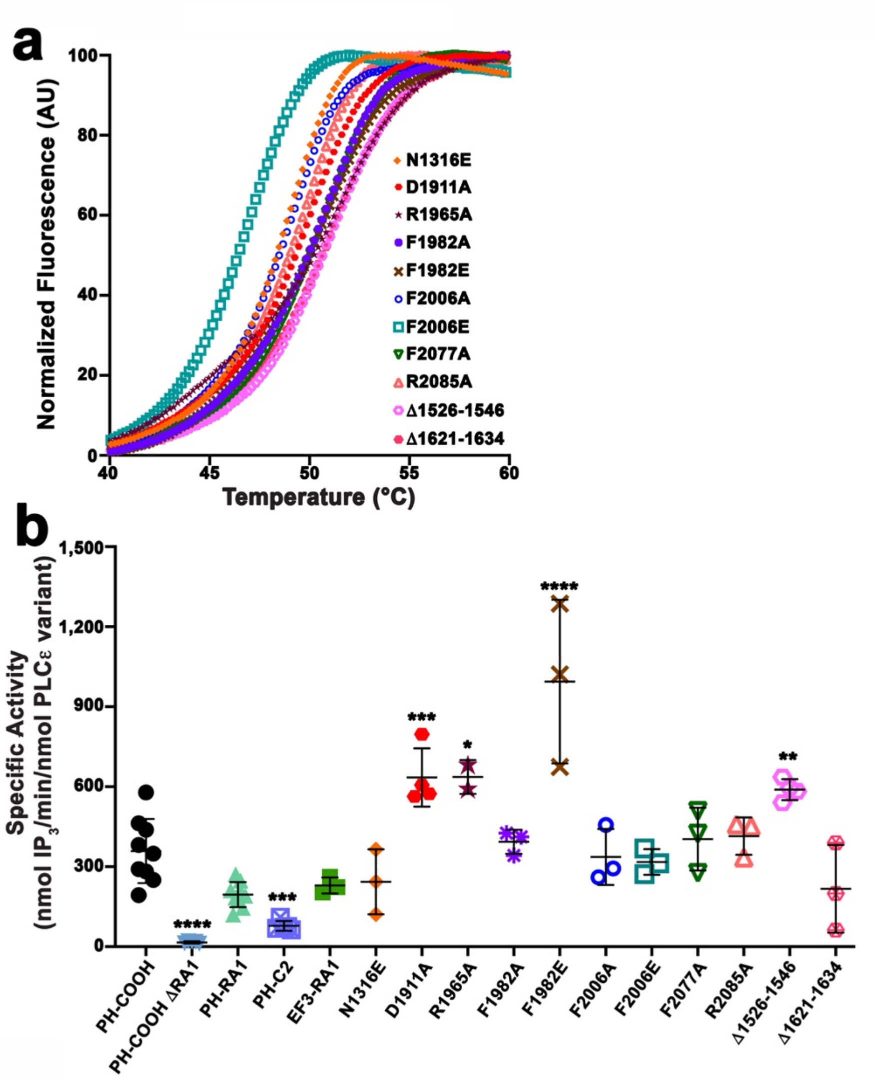

Supplementary Figure 8. Thermal stability and basal activity of PLCε variants. (A) Representative DSF curves of the PLCε PH-COOH point mutants and X–Y linker deletion variants. The T_m for each variant is determined from the inflection point of the denaturation curve. (B) Scatter plot of the specific activity of the PLCε variants. Error bars represent SD. Significance was determined based on one-way ANOVA followed by Dunnett's multiple comparisons test vs. PLCε PH-COOH (****, $p \leq 0.0001$, ***, $p \leq 0.0005$, **, $p \leq 0.001$, * $p \leq 0.05$). Data for PLCε PH-COOH and PH-C2 was previously published[22].

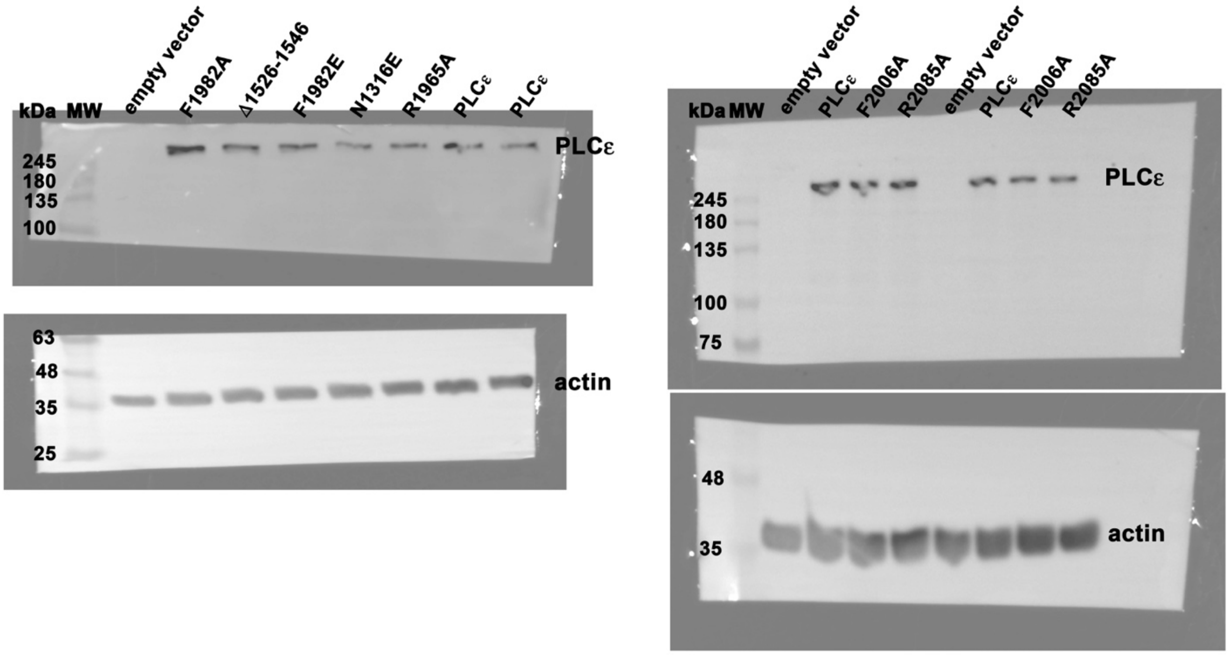

Supplementary Figure 9. Representative western blots of PLCε variants. PLCε variant expression in COS-7 cells was visualized and quantified by western blot, using empty vector as a negative control and actin as a loading control. The blots shown are the same as those shown in Figure 3C, but are not cropped. Cell lysates were divided into two pools, and analyzed on separate SDS-PAGE gels and western blots, due to the large size and technical difficulties in transferring the PLCε variants to the PVDF membrane. To minimize the amount of antibody needed for detecting the PLCε variants, the bottom one-third of the membrane was removed prior to incubation with the primary antibody.

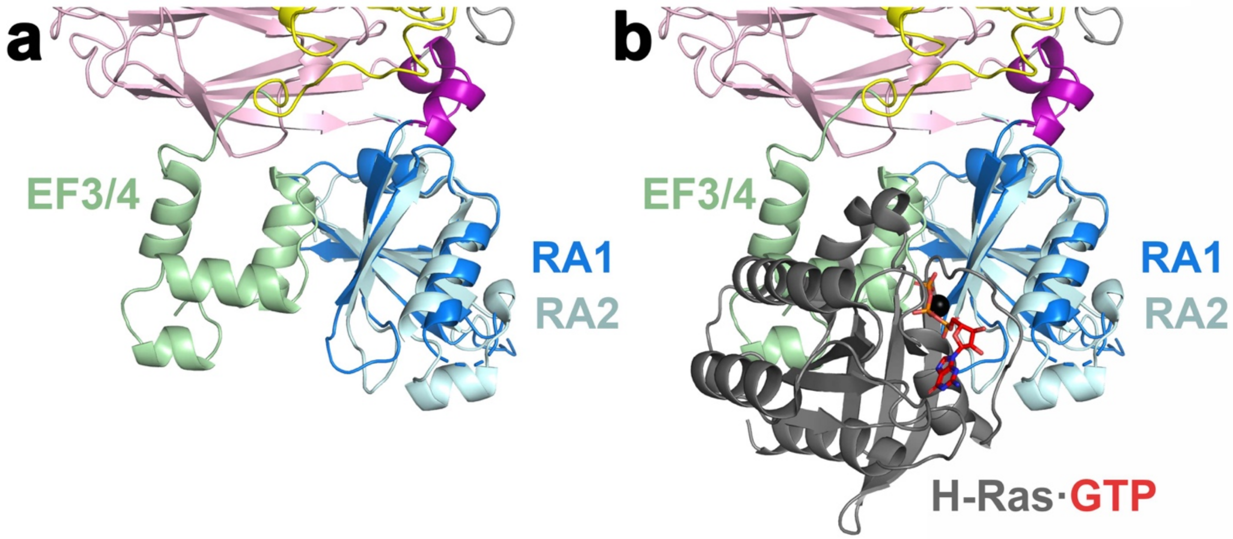

Supplementary Figure 10. The EF3/4–RA1 interface blocks small GTPase binding. (A) The structure of the RA1 domain in the PLCε EF3-RA1 structure is similar to the structure of the RA2 domain in complex with activated H-Ras (r.m.s.d. of 1.2 Å for 37 Cα, PDB ID 2C5L[18]). However, the PLCe RA1 domain is not known to contribute to GTPase binding. (B) Superimposing RA1 from the EF3-RA1 structure with RA2 in the H-Ras–RA2 structure shows that the vestigial GTPase binding surface on RA1 is blocked by EF3/4. H-Ras is shown in dark grey, Mg^{2+} as a black sphere, and GTP in red sticks.

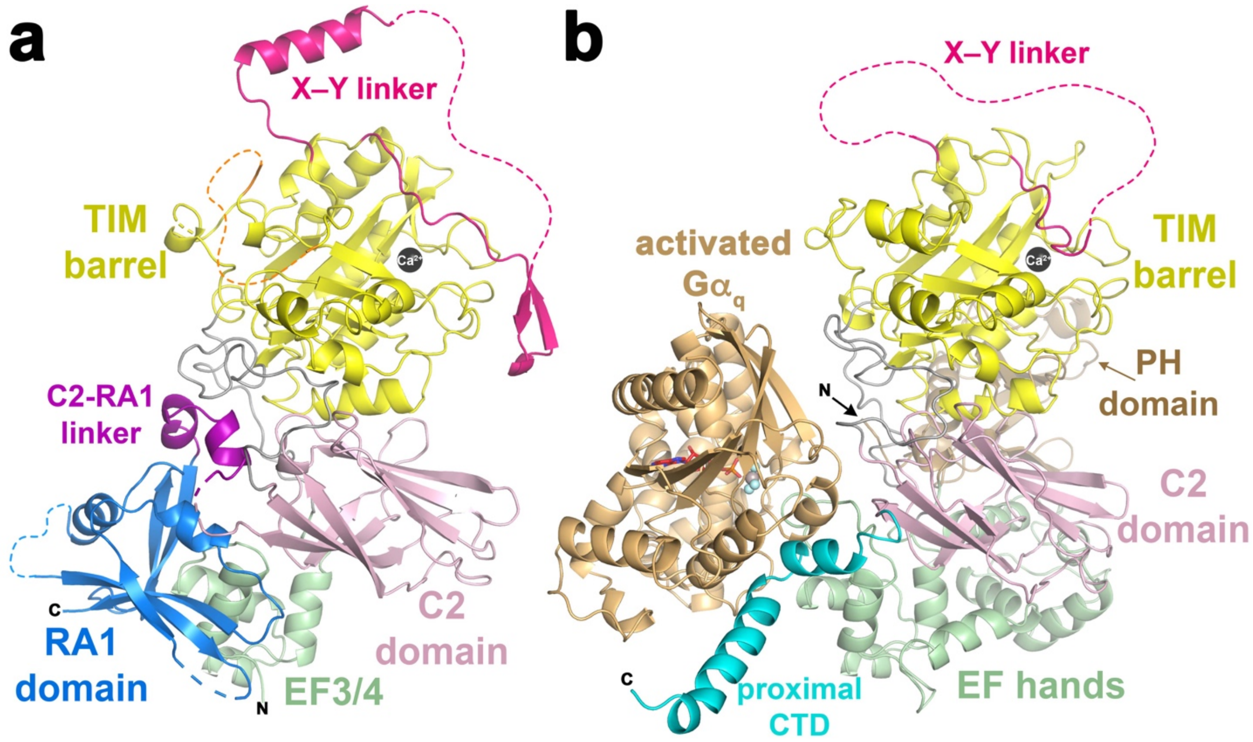

Supplementary Figure 11. PLCε and PLCβ may share a conserved regulatory interface. The TIM barrel-C2 domain interface may be a conserved regulatory hot spot in some PLC enzymes. (A) In the PLCε EF3-RA1 structure, the C2-RA1 linker docks at the TIM barrel-C2 interface. Deletion of this element, or mutations within this region decrease stability of the enzyme, but increase basal activity across assay formats (Figures 1, 4, Table 1). (B) The crystal structure of the PLCβ3 core and proximal C-terminal domain (CTD) in complex with the activated heterotrimeric G protein subunit $G\alpha_q$ (PDB ID 3OHM[39]). $G\alpha_q$ forms extensive interactions with the proximal CTD, EF3/4, and the C2 domain. These interactions occur on the same face of the PLC core as the C2-RA1 linker in the PLCε EF3-RA1 structure. Activated $G\alpha_q$ is shown in tan, with Mg^{2+} shown in a black sphere, GTP shown in red sticks, and AlF_4^- in tan and light blue spheres.

Supplementary Table 1. SAXS parameters of PLCε EF3-RA1

	**PLCε EF3-RA1**
Guinier analysis	
I(0) (Arb.)	113 ± 0.4
R_g (Å)	35 ± 2.0
q min ($Å^{-1}$)	0.0000805
q range ($Å^{-1}$)	0.0000805-0.00139
P(r) analysis	
I(0) (Arb.)	114
R_g (Å)	36
D_{max} (Å)	126
Porod volume ($Å^{-3}$)	152,000
q range ($Å^{-1}$)	0.0000805-0.230

Supplementary Table 2. SAXS Data Collection and Analysis Parameters

(a) SAXS data collection parameters	
Instrument	BioCAT facility at the Advanced Photon Source beamline 18ID with Pilatus3 X 1M (Dectris) detector
Wavelength (Å)	1.033
Beam size (μm²)	160 (h) x 75 (v)
Camera length (m)	3.7
q-measurement range (Å⁻¹)	0.004-0.4
Absolute scaling method	N/A or Glassy Carbon, NIST SRM 3600
Basis for normalization to constant counts	To incident intensity, by ion chamber counter
Method for monitoring radiation damage	Automated frame-by-frame comparison of relevant regions using CORMAP[5] implemented in BioXTAS RAW
Exposure time, number of exposures	0.5 s exposure time with 2 s exposure period (0.5 s on, 1.5 s off) of entire SEC elution
Sample configuration	SEC-SAXS. Separation by size using ÄKTA Pure with a Superdex 200 Increase 10/300 GL column. SAXS data was measured in a 1.5 mm ID quartz capillary with effective path length 0.542 mm.
Sample temperature (˚C)	20
(b) Software employed for SAXS data reduction, analysis and interpretation	
SAXS data reduction	Radial averaging; BioXTAS RAW 1.4.0[50] and ATSAS[54] used for frame comparison, averaging, and subtraction
Basic analysis: Guinier, M.W., P(r)	BioXTAS RAW 1.4.0[50] used for Guinier fit and molecular weight; GNOM[55] used for P(r) function
