# Supplementary material for: Structure of phospholipase Cε reveals an integrated RA1 domain and previously unidentified regulatory elements
Source: Commun Biol. 2020 Aug 14;3:445. doi: 10.1038/s42003-020-01178-8 (PMC7427993; doi:10.1038/s42003-020-01178-8)
Supplement: Supplementary file 2 — Reporting Summary [file 42003_2020_1178_MOESM2_ESM.pdf]

nature research

Corresponding author(s): Angeline M. Lyon

Last updated by author(s): Jun 24, 2020

Reporting Summary

Nature Research wishes to improve the reproducibility of the work that we publish. This form provides structure for consistency and transparency in reporting. For further information on Nature Research policies, see Authors & Referees and the Editorial Policy Checklist.

Statistics

For all statistical analyses, confirm that the following items are present in the figure legend, table legend, main text, or Methods section.

n/a	Confirmed	
☐	☒	The exact sample size (n) for each experimental group/condition, given as a discrete number and unit of measurement
☐	☒	A statement on whether measurements were taken from distinct samples or whether the same sample was measured repeatedly
☐	☒	The statistical test(s) used AND whether they are one- or two-sided<br>*Only common tests should be described solely by name; describe more complex techniques in the Methods section.*
☒	☐	A description of all covariates tested
☐	☒	A description of any assumptions or corrections, such as tests of normality and adjustment for multiple comparisons
☐	☒	A full description of the statistical parameters including central tendency (e.g. means) or other basic estimates (e.g. regression coefficient) AND variation (e.g. standard deviation) or associated estimates of uncertainty (e.g. confidence intervals)
☒	☐	For null hypothesis testing, the test statistic (e.g. F, t, r) with confidence intervals, effect sizes, degrees of freedom and P value noted<br>*Give P values as exact values whenever suitable.*
☒	☐	For Bayesian analysis, information on the choice of priors and Markov chain Monte Carlo settings
☒	☐	For hierarchical and complex designs, identification of the appropriate level for tests and full reporting of outcomes
☒	☐	Estimates of effect sizes (e.g. Cohen's d, Pearson's r), indicating how they were calculated

Our web collection on statistics for biologists contains articles on many of the points above.

Software and code

Policy information about availability of computer code

Data collection	X-ray diffraction data was collected using JBluIce available at the GM/CA beamline at the Advanced Photon Source (Sector 23). SAXS data was collected using BioXTAS-RAW 1.4.0.
Data analysis	Diffraction data was indexed, integrated, and scaled using autoPROC. The structure was solved by molecular replacement using PHASER 2.7.0. The final structural model was generated using alternating rounds of manual building in COOT 0.8 and refined in Refmac 5.5. Stereochemical correctness was assessed using Molprobity and Procheck. SAXS data was processed and analyzed using BioXTAS-RAW 1.4.0. All assay data was analyzed in GraphPad Prism v.8.0.1. Western blots were imaged and quantified with ImageJ.

For manuscripts utilizing custom algorithms or software that are central to the research but not yet described in published literature, software must be made available to editors/reviewers. We strongly encourage code deposition in a community repository (e.g. GitHub). See the Nature Research guidelines for submitting code & software for further information.

Data

Policy information about availability of data

All manuscripts must include a data availability statement. This statement should provide the following information, where applicable:

- Accession codes, unique identifiers, or web links for publicly available datasets
- A list of figures that have associated raw data
- A description of any restrictions on data availability

The structure factors and coordinates have been deposited as PDB ID 6PMP in the Protein Data Bank, and will be released upon publication. Plasmids and other reagents are available upon reasonable request to A.M.L.

Field-specific reporting

Please select the one below that is the best fit for your research. If you are not sure, read the appropriate sections before making your selection.

☒ Life sciences ☐ Behavioural & social sciences ☐ Ecological, evolutionary & environmental sciences

For a reference copy of the document with all sections, see nature.com/documents/nr-reporting-summary-flat.pdf

Life sciences study design

All studies must disclose on these points even when the disclosure is negative.

Sample size	No sample-size calculation was performed, as is standard for the field. All experiments were performed at least two times in duplicate. Statistical significance was used to determine sample size required.
Data exclusions	Automatic data exclusion was used during GraphPad Prism fitting.
Replication	All experiments were performed at least two times in duplicate, and findings were replicated through these means.
Randomization	Randomization is not relevant to this study because no human or animal subjects were used.
Blinding	Blinding was not relevant to this study because there was no random group allocation and all data was independently analyzed in the same manner

Reporting for specific materials, systems and methods

We require information from authors about some types of materials, experimental systems and methods used in many studies. Here, indicate whether each material, system or method listed is relevant to your study. If you are not sure if a list item applies to your research, read the appropriate section before selecting a response.

Materials & experimental systems

n/a	Involved in the study	
☐	☒	Antibodies
☐	☒	Eukaryotic cell lines
☒	☐	Palaeontology
☒	☐	Animals and other organisms
☒	☐	Human research participants
☒	☐	Clinical data

Methods

n/a	Involved in the study	
☒	☐	ChIP-seq
☒	☐	Flow cytometry
☒	☐	MRI-based neuroimaging

Antibodies

Antibodies used	mouse anti-FLAG and mouse anti-actin (Cell Signaling Technology)
Validation	Xu M, et al Interaction of YAP1 and mTOR promotes bladder cancer progression. Int J Oncol. 2020;56(1):232-242. doi:10.3892/ijo.2019.4922; Yao, D. et al J Enz Inhib Med Chem 35(1):713-725. doi.org/10.1080/14756366.2020.1740924 ; validation data on manufacturer's website (Cell Signaling Technology)

Eukaryotic cell lines

Policy information about cell lines

Cell line source(s)	COS-7 cells were a gift from A.V. Smrcka (U. Michigan), Sf9 cells were purchased from Expression Systems
Authentication	The COS-7 cells were not authenticated, and the Sf9 cells were not authenticated as they were purchased directly from the vendor.
Mycoplasma contamination	The COS-7 cells tested negative for mycoplasma contamination.
Commonly misidentified lines (See ICLAC register)	N/A
